# Dynamics and Trends in Fecal Biomarkers of Gut Function in Children from 1–24 Months in the MAL-ED Study

**DOI:** 10.4269/ajtmh.16-0496

**Published:** 2017-02-08

**Authors:** Benjamin J. J. McCormick, Gwenyth O. Lee, Jessica C. Seidman, Rashidul Haque, Dinesh Mondal, Josiane Quetz, Aldo A. M. Lima, Sudhir Babji, Gagandeep Kang, Sanjaya K. Shrestha, Carl J. Mason, Shahida Qureshi, Zulfiqar A. Bhutta, Maribel Paredes Olortegui, Pablo Peñataro Yori, Amidou Samie, Pascal Bessong, Caroline Amour, Estomih Mduma, Crystal L. Patil, Richard L. Guerrant, Dennis R. Lang, Michael Gottlieb, Laura E. Caulfield, Margaret N. Kosek

**Affiliations:** 1 Fogarty International Center/National Institutes of Health, Bethesda, Maryland.; 2 Tulane University, New Orleans, Louisiana.; 3 International Centre for Diarrhoeal Disease Research, Bangladesh (icddr,b), Dhaka, Bangladesh.; 4 Universidade Federal do Ceara, Fortaleza, Brazil.; 5 Christian Medical College, Vellore, India.; 6 Walter Reed/Armed Forces Research Institute of Medical Science (AFRIMS) Research Unit, Kathmandu, Nepal.; 7 Aga Khan University, Karachi, Pakistan.; 8 Asociacion Benéfica Proyectos en Informatica, Salud, Medicina, y Agricultura (A. B. PRISMA), Iquitos, Peru.; 9 University of Venda, Thohoyandou, South Africa.; 10 Haydom Lutheran Hospital, Haydom, Tanzania.; 11 University of Illinois at Chicago, Chicago, Illinois.; 12 University of Virginia, Charlottesville, Virginia.; 13 Foundation for the National Institutes of Health, Bethesda, Maryland.; 14 Johns Hopkins Bloomberg School of Public Health, Baltimore, Maryland.

## Abstract

Growth and development shortfalls that are disproportionately prevalent in children living in poor environmental conditions are postulated to result, at least in part, from abnormal gut function. Using data from The Etiology, Risk Factors, and Interactions of Enteric Infections and Malnutrition and the Consequences for Child Health and Development (MAL-ED) longitudinal cohort study, we examine biomarkers of gut inflammation and permeability in relation to environmental exposures and feeding practices. Trends in the concentrations of three biomarkers, myeloperoxidase (MPO), neopterin (NEO), and α-1-antitrypsin (AAT), are described from fecal samples collected during the first 2 years of each child's life. A total of 22,846 stool samples were processed during the longitudinal sampling of 2,076 children 0–24 months of age. Linear mixed models were constructed to examine the relationship between biomarker concentrations and recent food intake, symptoms of illness, concurrent enteropathogen infection, and socioeconomic status. Average concentrations of MPO, NEO, and AAT were considerably higher than published references for healthy adults. The concentration of each biomarker tended to decrease over the first 2 years of life and was highly variable between samples from each individual child. Both MPO and AAT were significantly elevated by recent breast milk intake. All three biomarkers were associated with pathogen presence, although the strength and direction varied by pathogen. The interpretation of biomarker concentrations is subject to the context of their collection. Herein, we identify that common factors (age, breast milk, and enteric infection) influence the concentration of these biomarkers. Within the context of low- and middle-income communities, we observe concentrations that indicate gut abnormalities, but more appropriate reference standards are needed.

## Introduction

In recent years, researchers have proposed that a significant cause of growth faltering and malnutrition, which disproportionately occurs early in life in children residing in low- and middle-income countries (LMICs), is due to changes in gut function resulting from environmental exposures to enteropathogens and toxins, from inadequate nutrient intake, and from disease history, notably diarrhea.^1^,^2^ Various alterations in gut function have been associated with environmental enteropathy (EE), a condition histopathologically defined from examination of biopsy material characterized by changed morphology (crypt depth and villus height) and altered inflammatory and immune responses.^1^,^3^ In an effort to move to noninvasive diagnostics for EE, various biomarkers of gut and systemic inflammation and gut permeability have been proposed and studied. The goal of such studies is to better characterize specific changes that are predictive of, and causally related to, the aforementioned physical growth and related developmental shortfalls.^3^,^4^

The Etiology, Risk Factors, and Interactions of Enteric Infections and Malnutrition and the Consequences for Child Health and Development (MAL-ED) study evaluated three readily collectable fecal biomarkers of gut immunity and permeability: myeloperoxidase (MPO) to indicate neutrophil activity in the intestinal mucosa, neopterin (NEO) an indicator of T-helper cell 1 activity, and alpha-1-antitrypsin (AAT) to indicate intestinal permeability and protein loss.^4^,^5^ These markers have been used to assess gastrointestinal health in the context of chronic disease (e.g., inflammatory bowel disease [IBD]), but have not been studied fully in children, and there are no established thresholds for what might be considered healthy values or indicative of abnormality. However, fecal concentrations of these biomarkers have been shown to be directly associated with the number of pathogens detected in the stool, and most strongly with pathogens that are enteroinvasive or those that cause mucosal disruption.^6^ Poor sanitation and hygiene conditions associated with lower socioeconomic status (SES) are associated with higher concentrations of these (significantly higher MPO concentrations in children of lower SES within the same population; G. Kang, personal communication) and related markers of gut function,^7^ supporting earlier histopathology studies, suggesting that environmental conditions rooted in poverty may lead to subsequent detrimental outcomes.^8^ In both MAL-ED and in other studies where growth faltering is common, these biomarkers have been shown to be inversely associated with concurrent or future measures of child growth.^6^,^9^,^10^

What is unclear, however, is whether and to what extent these biomarkers are additionally stimulated by factors besides enteropathogens. Breast milk is known to contain proteins (including AAT and NEO) that influence gut growth and immune development as well as the development of microbial communities.^11^,^12^ Non-breast-milk food intake and digestion also stimulate peristalsis, increase stool volume, and alter microbial communities affecting permeability. Thus, it may be that characteristics of the infant's diet influence the concentrations of these biomarkers. Similarly, infections at distant mucosal sites may alter mucosal permeability, which may in turn affect intestinal inflammation. A better understanding of the role that such factors play in affecting the fecal concentrations of MPO, AAT, and NEO may be important to evaluate their utility as predictive or causal biomarkers and/or for establishing parameters for sampling or interpretation.

Herein, we use longitudinal surveillance data from MAL-ED to evaluate trends over time in these three fecal biomarkers of gut inflammation and permeability, and evaluate household, seasonal, maternal, and child-level factors affecting their distributions.

## Materials and Methods

### Data.

Detailed elsewhere, the MAL-ED study is a longitudinal cohort study conducted in eight populations: Dhaka, Bangladesh; Fortaleza, Brazil; Vellore, India; Bhaktapur, Nepal; Loreto, Peru; Naushero Feroze, Pakistan; Venda, South Africa; and Haydom, Tanzania.^13^

In brief, children were enrolled within 17 days of birth, but excluded if: their birth weight was < 1,500 g, were very ill, or non-singleton; their mother was < 16 years of age; or the families planned to leave the area within 6 months. The goal was to follow 200 children to 24 months of age, thus, ≥ 200 children were recruited to account for loss to follow up at each site. The methods of data collection for infant feeding,^14^ disease surveillance,^15^ and stool microbiology^16^ are described elsewhere. Each site obtained ethical approval from their respective institutions, and written consent was obtained from participants.

At enrollment, the date of birth, sex, and early feeding experiences were recorded, as was birth weight if available. Thereafter, through twice-weekly surveillance visits, caregivers were asked about breastfeeding and non-breast-milk food consumption.^14^ The presence or absence of specific foodstuffs (including animal milk, formula, juice, teas, grains, roots/tubers, fruit) was also recorded. Mothers also reported whether the child had illness in the preceding days including diarrhea, fever, or respiratory symptoms (which were used in conjunction with fieldworker-measured rapid respiration rate to identify acute lower respiratory infection [ALRI]).^15^ Anthropometric measures were taken by trained personnel at enrollment and monthly to 24 months of age.^17^ Monthly visits for anthropometry and data collection were scheduled for the child's birthday each month.

A questionnaire to assess household SES was administered every 6 months,^18^ but since factors were invariant over time, the mean (continuous) or mode (categorical) average responses were calculated. Questions were asked of each household concerning the construction of the house, the monthly income, maternal education, and water and sanitation. These were combined into a single index (water and sanitation, assets, maternal education, and income [WAMI]) that was inversely related to stunting within pilot data and described the range of SES both between and within sites.^18^

Normal stools were collected for analysis monthly during the first year and quarterly in the second. Additional stools were collected when the mother reported diarrhea (≥ 3 loose stools within a 24-hour period) during the biweekly morbidity surveillance visits. Three biomarkers were analyzed in the monthly stool samples: 1) MPO (ng/mL) (Alpco, Salem, NH); 2) NEO (nmol/L) (GenWay Biotech, San Diego, CA); and 3) AAT (μg/g) (Biovendor, Candler, NC). In each case, the kit protocols for biomarker assays were followed using 1:500 dilution for MPO and AAT and 1:1,000 in 0.9% saline for NEO, having collected samples without fixative and storing them at −70°C before analysis.^4^ Dilutions were increased or decreased as required for out-of-range samples. Because biomarkers were assessed as wet volume, a qualitative description of liquidity of each stool sample was noted in the receiving laboratory.

Stools were excluded from the analysis if they were collected outside the window of the sampling schedule (−2 ≤ days ≤ 7). Stool samples were additionally excluded if diarrhea was reported in the 7 days preceding the stool collection because diarrhea leads to stool dilution. Stools collected the same or following day of a lactulose:mannitol (L:M) test were also excluded as lactulose is an osmotic laxative.^9^

Each stool sample was subjected to a panel of tests for approximately 40 pathogens as previously described.^16^ In addition to concurrent enteropathogens, the cumulative average number of different enteropathogens detected per stool was calculated (i.e., up to the age of a given stool collection).

### Statistics.

The concentrations of all three biomarkers were highly skewed, and therefore, each was log-transformed to make them approximately normally distributed. To evaluate potential associations between biomarkers, Spearman's rank correlations were calculated at each month of age.

Linear mixed-effects models, accounting for repeated measures of the same child, were constructed to evaluate factors that affect biomarker concentration. A single model was made for each of the three biomarkers. To account for clustering in the variance, random slopes and intercepts were included for each child and each site (selecting between random structures using Akaike information criteria). Permutations of how to combine variables were assessed (see Supplemental Information) and the optimal model, based on AIC, included a cubic spline for the age term and interactions between the domain variables and age. Knots were included at 6 and 12 months to account for both a flexible age trend and changes in feeding practice over the first 2 years. In each model, the pathogens in the same stools as the biomarker assays, binary variables for whether the child received breast milk or had any reported illness (diarrhea, ALRI, or fever) in the preceding 7 days, and the WAMI index and enrollment weight-for-age *z* score were included. Seasonality in the biomarkers was assessed using a partial Fourier series with annual and biannual harmonics. Given that sites could have very different seasonal patterns, these were included only as the interaction terms with site (i.e., main effects for the harmonics were not included). Goodness of fit for the final model was measured with marginal and conditional *r*^2^,^19^ and the proportion of variance explained by individual variables was calculated using analysis of variance.

## Results

**Figure 1.**
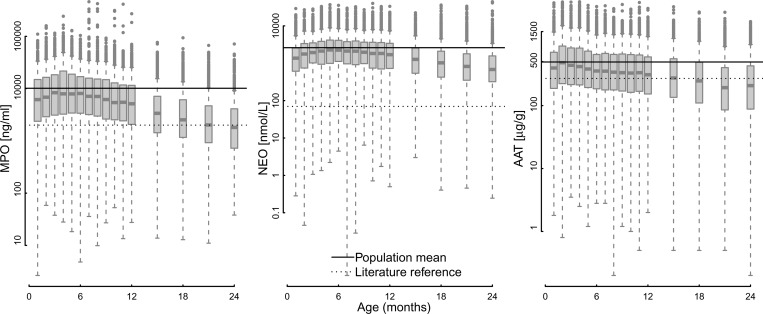
Distributions of the pooled log myeloperoxidase (MPO), neopterin (NEO), and α-1-antitrypsin (AAT) concentrations by month of age. Solid horizontal lines indicate the mean observed value. Dashed lines indicate values reported from in the literature^9^,^10^: MPO < 2,000 ng/mL^20^; NEO < 70 nmol/L^21^; and AAT < 270 μg/g.^22^

All three biomarkers had high average concentrations relative to published references for healthy individuals in high-income country settings (Figure 1

, Table 1). Within MAL-ED, the geometric mean concentration of MPO was 4,815 ng/mL, NEO 1,372 nmol/L, and AAT 299 μg/g compared with values reported in the literature of 2,000 ng/mL,^20^ 70 nmol/L,^21^ and 270 μg/g, respectively.^22^ The log concentration of all three biomarkers decreased as children aged (Figure 1), and average values differed among the eight populations (Supplemental Figures 1–4). By the second year, average MPO concentrations tended to converge on published concentrations, whereas the other two fecal biomarkers remained substantially higher on average. Natural cubic spline terms for age accounted for 11.8% of MPO, 5.4% of NEO, and 5.3% of AAT variance. The within-child variability was high with child-level intraclass correlations of 0.11, 0.06, and 0.19 for MPO, NEO, and AAT, respectively, indicating only moderate within-child clustering of the data. The three biomarkers were also only weakly correlated with one another (< 0.25 Spearman's correlation coefficient), so that elevation of one biomarker would not necessarily correspond with elevated concentrations of the other two. Given that diarrheal stools were more liquid and this altered the measured concentrations of biomarkers (Supplemental Table 1), diarrheal stools were excluded from analysis.

Child weight for age at enrollment was not statistically associated with either MPO or NEO concentrations; however, it was significantly positively associated with the concentration of AAT (i.e., heavier children had higher AAT concentrations on average).

**Figure 2.**
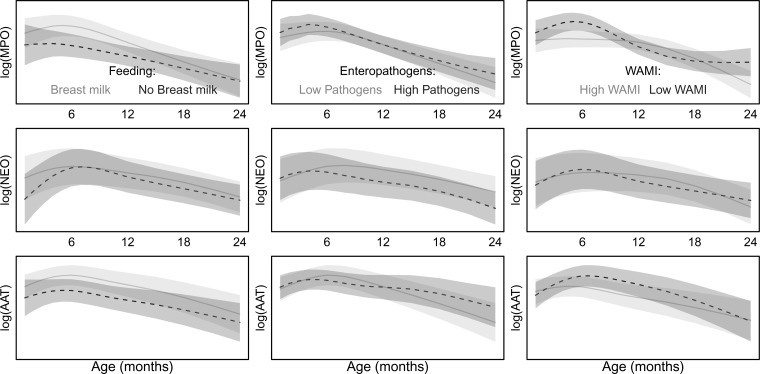
Predicted concentrations of each fecal biomarker in the presence or absence of a single factor while keeping all other predictors equal. (left column) Effects of breast milk in the 7 days preceding the stool collection, with higher biomarker concentrations association with the presence of breast milk (3–12 months for myeloperoxidase [MPO] and α-1-antitrypsin [AAT]); (middle column) low (5th percentile) or high (95th percentile) cumulative pathogen detections, indicating slightly higher biomarker concentrations during the first and last 6 months for MPO associated with higher pathogen detections and in the second year of life for AAT. neopterin [NEO] is lower with higher cumulative pathogens detected; (right column) and the effect of low (5th percentile) or high (95th percentile) socioeconomic index (water and sanitation, assets, maternal education, and income [WAMI]) on biomarker concentrations and how these effects relate to the age of a child with higher socioeconomic status associated with lower MPO and AAT biomarker concentrations. Grey areas show the 95% confidence interval for the mean prediction of fixed effects (conditional on the site and child random effects).

The associations between both the concurrent enteropathogens detected in the same stools assayed for the fecal biomarkers and the cumulative average number of different pathogens detected per stool leading up to a given biomarker assay were examined. All three biomarkers were better described (comparing AICs of the alternative models) by the cumulative pathogen burden than the single contemporary pathogens detected. A higher average number of pathogens per stool was associated with a small, but increased concentration of MPO. Given the age trend in the relationship, on average, this resulted in increased MPO during the first 6 months and in the second year of life. The mean concentration of AAT was similarly increased in the second year of life by higher average pathogen burden (Figure 2

). Though the main effect of enteropathogen detections was to increase NEO concentration, the net effect including interaction terms with age was a decrease in NEO concentrations (Figure 2). The most commonly detected pathogen, *Campylobacter*, was consistently and positively associated with MPO concentration across all eight sites. *Giardia* was positively associated with NEO in six sites (Supplemental Figure 6).

Symptoms of diarrhea, fever, or ALRI contributed little to the models (< 1% of variance explained for all three models). In the second year, recent illness was associated with an increase in NEO (0.3 ± 0.06, mean ± standard deviation) but a decrease in MPO (−0.3 ± 0.07).

**Figure 3.**
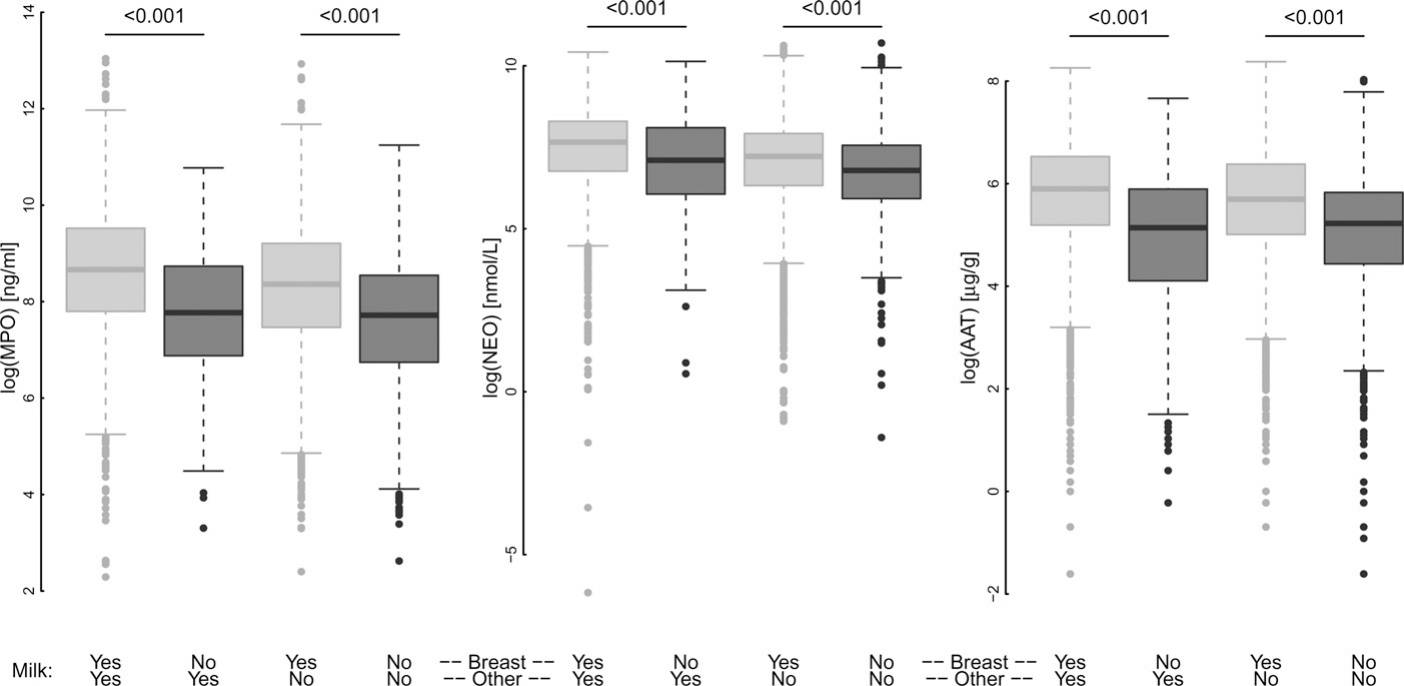
The concentration of fecal biomarkers in children 7–24 months (pooled across sites) for children receiving breast or other (animal or formula) milks in the 7 days preceding the stool collection. Comparison between those children who received breast milk and those who received alternative sources of milk was made using Wilcox signed rank tests.

Consumption of breast milk was nearly universal in the first 6 months (98% of children had some breast milk in the 7 days before stool collection) and decreased to 61% in the second year (pooling all sites, Table 1, Supplemental Figure 5). Breast milk consumption in the 7 days preceding the assay was associated with increased concentrations of MPO and AAT. Differences in concentration between those children with or without any recent breast milk intake were largest at 7–12 months of age for all three biomarkers (Figure 2). Although children frequently received animal milks (replacing or in addition to breast milk) starting at 3 months of age, only breast milk intake was associated with higher biomarker concentrations (Figure 3

). The influence of specific types of solid foods on the biomarkers was variable by site (Supplemental Figure 6), reflecting the variability in weaning foods within and across the sites.

The concentrations of MPO and AAT were higher with lower socioeconomic WAMI score (Figure 2). This was especially pronounced for MPO and in particular during the first 6 months of life. WAMI explained the second largest proportion of variance (2.8, 6.1, and 0.4% of MPO, NEO, and AAT, respectively) after the age terms. The WAMI score aggregates across multiple domains; however, none of the individual components were consistently associated across sites in the same way as the overall score (Supplemental Figure 6). Of the domains of WAMI, income was informative to describe variability in AAT concentrations for several sites, as was the location of drinking water wherein children from households with water in the dwelling had significantly lower concentrations of AAT than those that did not. Perhaps surprisingly, neither floor nor toilet type were informative in the site models though this may be because water, sanitation, and hygiene factors were better captured by the overall WAMI score and enteropathogen detections (Supplemental Figure 6).

There were small, but significant site-specific seasonal patterns in all three biomarkers accounting for 1.8%, 1.7%, and 1.4% of MPO, NEO, and AAT variance, respectively (Supplemental Figure 7). These effects were detectable after adjustment for concurrent and cumulative presence of enteropathogens and recent current infant feeding.

The explanatory power of the models of pooled data suggested that the fixed effects, while statistically significant, explained little variability (marginal *r*^2^ of 0.07 NEO, 0.08 AAT, and 0.16 MPO). The inclusion of random intercepts and slopes per child and site substantially increased the variance explained (conditional *r*^2^ of 0.34 NEO, 0.29 AAT, and 0.30 MPO). This suggests that a large proportion of the variance in the biomarkers could not be explained by any of the factors included in our model and that variability in observed concentrations was attributable to other child or environmental factors than those examined.

## Discussion

There is a need for noninvasive biomarkers to assess intestinal inflammation and permeability in young children. Herein, we have evaluated three commercially available biomarkers that have a demonstrable track record in assessing chronic disease of the gut (e.g., IBD, Crohn's or celiac diseases). The use of MPO, NEO, and AAT to evaluate gut permeability and immunity in infants and children growing up in LMICs is novel, and we have assessed them in eight populations with some 16 measures per child over the first 2 years of life. Herein, we present analyses on the nature of the distributions and how they change over time and identify factors associated with their concentrations in easily obtained normal stool samples.

Infancy is a period of rapid gastrointestinal development, including age-related physiological changes such as “gut closure” (a process by which intestinal permeability in the newborn rapidly declines during the neonatal period),^24^,^25^ which is also accompanied by marked age-related changes in the host immune system and the intestinal microbiota.^26^–^28^ The results we present herein show how concentrations of fecal biomarkers of gut permeability and inflammation change substantially over the first 2 years of life. Declines are noted in each of the three biomarkers, yet only in the second year of life did average values for MPO approach published reference values.

The elevated concentrations relative to reference data may be partially explained by continued consumption of breast milk throughout the second year of life for most infants and the positive association between breast milk in the diet and concentrations of MPO and AAT. Breast milk contains AAT, with higher concentrations (0.3 g/L) found in colostrum and lower, stable concentrations (0.1 g/L) in mature milk at 30 days,^29^ which coincides with the first stool collection. Although less well characterized, it is thought that human milk contains pico-quantities per liter of NEO, and even smaller quantities are found in various animal milks and formula.^30^ Given milk volumes on the order of 0.7 L/day during the first 6 months,^31^ only a small fraction of the AAT and NEO detected in these stools is of maternal origin. Breast milk is also known to contain neutrophils, yet they are thought to have a role in maternal immunity alone,^11^ and the amount of MPO in breastmilk is not well described. However, breast milk contains alpha-lactalbumin, a tryptophan-rich protein, and both tryptophan itself and digestive protein fragments from alpha-lactalbumin have been found to activate infant mucosal neutrophils, leading to MPO production.^32^,^33^ In contrast to other causes of mucosal neutrophil activation, this process is thought to be adaptive. These points highlight the difficulty of evaluating permeability and immunity in breastfed children. Although some have argued that, for example, fecal AAT should not be used to evaluate enteropathy during infancy because breast milk contains AAT,^34^ we show positive associations between enteropathogen exposure and diarrheal illness and elevated concentrations adjusting for the presence of breast milk in the diet. Given that many children worldwide are breastfed into the second year of life and later, biomarker reference data would ideally be developed among breastfed children, and in the absence of that, adjusting for breastfeeding status as shown here would be recommended.

The association between enteropathogens and these biomarkers has been reported before, in particular *Campylobacter*, the most commonly detected pathogen in the MAL-ED study, with higher MPO concentrations.^6^ Although the number of pathogens in the same stools assayed for the biomarkers were associated with higher biomarker concentrations, the mean effect is greater when considering the cumulative average number of pathogens. EE has been attributed to frequent and high enteropathogen burden, and the positive relationship between biomarker concentrations and cumulative number of pathogens supports this hypothesis. The cumulative average number of pathogens not only captures repeated detections, but a high average number of pathogens also means little time without pathogens, which would be important for recovery of growth deficits.^35^,^36^ In contrast, a parallel analysis to identify influences on the L:M test as a measure of permeability in these children, found that variability in the L:M ratio or its components was associated with concurrent rather than longer term pathogen exposure.^37^ Thus, these different biomarkers of EE provide unique information toward our understanding of how enteropathogens disrupt gut function. The complex association between SES and MPO and AAT is concordant with previous reports that EE is a function of poverty.^38^ Low SES, when measured through access to improved water and sanitation services, has previously been used as a proxy for the frequency of pathogen challenge, and interpreted as the accumulation of insults over time.

It is noteworthy that none of the variables characterizing morbidity history (diarrhea, fever, or ALRI) explained very much of the variability in these biomarkers over time. These findings support current thinking that enteropathogen presence and inflammation in the gut is more important than the presence of epidemiologically defined diarrheal illness episodes on host physiology. They also suggest that the fecal biomarkers can be used in conjunction with biomarkers of systemic infection in studies of causal pathways linking inflammation with chronic undernutrition or other important health outcomes.^6^

Infants with a lower weight for age at enrollment (< 17 days) did not have different biomarker concentrations either at 1 month or throughout the 24-month follow-up. We do not have information on gestational age at delivery and cannot assess differences in biomarkers associated with preterm birth. We also excluded babies weighing < 1,500 g at birth from the study. However, our results generally indicate that the biomarker concentrations are not affected by size at birth.

This study spans multiple populations and work was conducted across local laboratories. An advantage of these biomarkers is that they can be evaluated using commercially available kits with internal standards and demonstrated high reproducibility. During the study period, ongoing quality control activities were also conducted to ensure that protocols were standardized across laboratories, The use of wet volume remains, however, a potential limitation. Unpublished data suggest that concentrations of these three biomarkers scales approximately linearly with water content in freeze-dried samples (G. O. Lee, personal communication) and standardizing by protein,^39^ but the verbal and postcollection classification of stool consistency was evidently an important predictor of concentration that might have been more precisely captured using dry weight or standardized dilution.

New biomarkers are being sought to indicate different aspects of gut immunity and permeability and these have promising results.^10^,^39^ The emerging conclusion of the ongoing search for noninvasive biomarkers of EE is that a panel of multiple biomarkers is superior to any one and that additional biomarkers may complement the three presented here.^9^,^10^ The challenge with each additional biomarker is, however, that they too are unlikely to be characterized in young children or ever been examined over time and thus subject to similar challenges of interpretation as the three discussed here. As these and other biomarkers are more clearly described, patterns may emerge that show more objective thresholds for comparison with groups of children in high-income countries or across a broader spread of SES than described here. As comparator data become available, explanations for some factors such as the variance attributed to seasonality that persisted, even accounting for recent food and pathogen exposure, may become evident.

In summary, the results presented here indicate that concentrations of fecal MPO, NEO, and AAT change over the first 24 months of life with higher values in the first year of life, declining thereafter. Concentrations were elevated among breastfed children, among those with higher rates of pathogen detection, and among those living in households of lower SES. Together, the findings support the use of these biomarkers to indicate intestinal inflammation and enteropathogen pressure in studies that cannot conduct detailed diagnostic panels to adequately characterize enteropathogen exposure. Prior values and cutoff points based on data from healthy adults in the developing world should not be used as reference values to assess gut health in children living in LMIC settings, and appropriate reference values among healthy children whose breastfeeding status and history of exposure to enteric pathogens is well characterized should be developed.

## Supplementary Material

Supplemental information, table, and figures.

## Figures and Tables

**Table 1:** Characteristics of the MAL-ED populations included for analysis

Variable	Age (months)				Range of site means
	0–6	7–12	13–24	Constant (i.e., all ages)	
No. of children	1,879	1,861	1,730		
No. of stools	7,470	8,300	5,021		
MPO (ng/mL)*	6,837 (3.6)	5,547 (3.5)	2,257 (3.6)		(2,552, 7,492)
NEO (nmol/L)*	1,595 (3.6)	1,585 (3.7)	853 (3.5)		(483, 3,393)
AAT (μg/g)*	378 (2.8)	310 (2.9)	199 (3.5)		(1,736, 429)
Pathogens/stool‡	0.08 (0, 0.21)	0.47 (0.29, 0.73)	0.92 (0.67, 1.23)		(0.24, 0.75)
Breast fed§	0.98	0.93	0.61		(0.78, 0.97)
Ill§	0.27	0.27	0.26		(0.32, 0.9)
Diarrhea§	0.01	0.02	0.02		(0, 0.05)
Fever§	0.13	0.14	0.13		(0.01, 0.34)
Antibiotics§	0.12	0.14	0.12		(0.02, 0.29)
Enrollment WAZ†				−0.69 (1.15)	(−1.6, 0.44)
WAMI†				0.57 (0.22)	(0.23, 0.83)
Household income (US$/month)‡				174.72 (80, 220.53)	(33.55, 354.18)
Improved sanitation¶**				0.7	(0.15, 1)
Improved drinking water¶**				0.89	(0.36, 1)

AAT = α-1-antitrypsin; MAL-ED = The Etiology, Risk Factors, and Interactions of Enteric Infections and Malnutrition and the Consequences for Child Health and Development Project; MPO = myeloperoxidase; NEO = neopterin; WAMI = water and sanitation, assets, maternal education, and income; WAZ = weight-for-age *z* score.

* Geometric mean (geometric standard deviation).

† Mean (standard deviation).

‡ Median (25th, 75th percentiles).

§ Proportion positive in the preceding 7 days.

¶ Proportion.

** Improved water and sanitation are based on World Health Organization definitions.^23^
