# Supplementary material for: Dynamics and Trends in Fecal Biomarkers of Gut Function in Children from 1–24 Months in the MAL-ED Study
Source: Am J Trop Med Hyg. 2017 Feb 8;96(2):465–72. doi: 10.4269/ajtmh.16-0496 (PMC5303054; doi:10.4269/ajtmh.16-0496)
Supplement: Supplementary file 1 [file SD11.pdf]

SUPPLEMENTAL INFORMATION

VARIABLE SELECTION

In a more detailed analysis, each site was modeled separately using finer-resolution variables to distinguish between the domain factors of diet, socioeconomic status, and intensity, and identification of enteropathogen exposures. The purpose of these models was to examine those local combinations of factors at each site that drive the population-specific observed concentrations. The detection of individual enteropathogens assessed in our diagnostic panel were included as candidate variables. The water and sanitation, assets, maternal education, and income (WAMI) score was broken down into a series of binary variables describing the source of drinking water and its location, type of toilet, type of stove, and the materials used for floor, walls, and ceiling. Additionally, continuous variables were included for the number of rooms in the household, the number of people sleeping there during the day, the household income (standardized to US$), and the maternal years of education. Food intake variables were broken down into different liquids and solids. Binary variables to indicate whether the child had experienced diarrhea, fever, vomiting, or had used antibiotics in the 7 days preceding the collection of the stool sample were included. Season was included as a variable to indicate the year quarter given that specific seasonal patterns may be different at each site (e.g., temperate or tropical).

Of the 102 candidate variables, many factors were absent or rare in a given site. A stochastic search variable selection[1,2] was implemented in JAGS[3] to identify variables that contributed to a final model per site. In brief, variable coefficients (β terms) in an additive model are estimated with a mixture of two normal distributions, namely a distribution describing the effect of the variable and a latent variable that describes the probability of retaining the variable.

The regression model takes the form

$$Y|\beta,\sigma^2 \sim N(X\beta,\sigma^2 I),$$

where observations of Y are a function of some set of predictors X multiplied by their coefficients β, with normally distributed error with variance σ^2. The β terms are then estimated by

$$\beta_i|\gamma_i \sim (1-\gamma_i)N(0,v_{ui}) + \gamma_i N(0,v_{ui}),$$

such that a binary latent variable γ for each ith predictor variable can be translated into a probability that a variable is kept. If γ_i is 0, then the β_i is drawn from a normal distribution with a small variance v_{ui}, and therefore likely to be approximated close to 0. However, where γ_i is 1, then the β_i is from a normal distribution with a much more diffuse variance v_{li}, and thus more likely to be estimated as non-zero.[4] Given the large number of variables (and thereby parameters) being examined, a simpler additive model with no interaction terms was fit.

SEASONALITY

Partial Fourier series were fit to the data using harmonics with an annual or biannual frequency (i.e., one or two peaks per year).

SUPPLEMENTAL REFERENCES

1. George EI, McCulloch RE, 1993. Variable selection via Gibbs sampling. *J Am Stat Assoc 88:* 881–889.
2. O'Hara RB, Sillanpää MJ, 2009. A review of Bayesian variable selection methods: what, how and which. *Bayesian Anal 4:* 85–117.
3. Plummer M, 2003. *JAGS: A Program for Analysis of Bayesian Graphical Models Using Gibbs Sampling*. The 3rd International Workshop on Distributed Statistical Computing (DSC 2003), March 20–22, 2003. Available at: http://www.ci.tuwien.ac.at/Conferences/DSC-2003/Drafts/Plummer.pdf. Accessed August 9, 2012.
4. Chipman H, George EI, McCulloch RE, Clyde M, Foster DP, Stine RA, 2001. The practical implementation of Bayesian model selection. *Lect Notes Monogr Ser 38:* 65–134.
5. Saiki T, 1998. Myeloperoxidase concentrations in the stool as a new parameter of inflammatory bowel disease. *Kurume Med J 45:* 69–73.
6. Ledjeff E, Artner-Dworzak E, Witasek A, Fuchs D, Hausen A, 2013. Neopterin concentrations in colon dialysate. *Pteridines 12:* 155–160.
7. Beckmann G, Rüffer A, 2000. *Mikroökologie des Darms: Grundlagen–Diagnostik–Therapie* (vergriffen, keine Neuauflage). Hannover, Germany: Schlütersche Verlag.

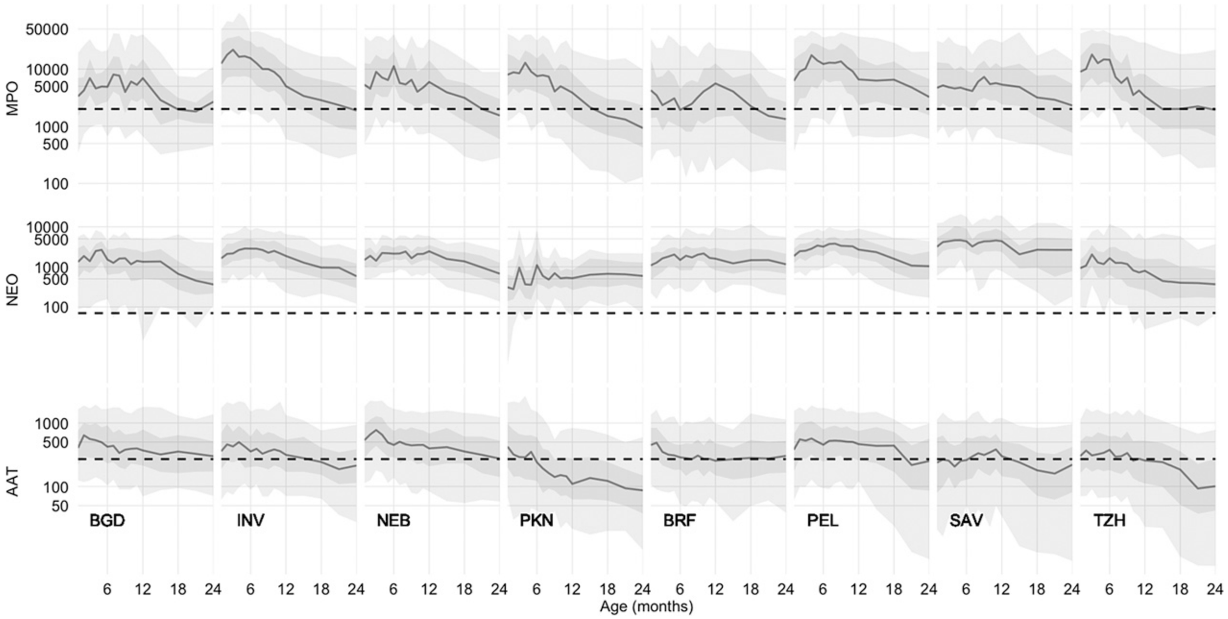

Supplemental Figure 1. The median (dark solid line) 25th–75th percentiles (dark grey) and 5th–95th percentiles (light grey) concentrations of myeloperoxidase (MPO; top), neopterin (NEO; middle), and α-1-antitrypsin (AAT; bottom row) for each MAL-ED site. The dashed horizontal lines indicate values reported from in the literature: MPO < 2,000 ng/mL[5]; NEO < 70 nmol/L[6]; and AAT < 270 μg/g.[7] Site-specific 95th percentiles are labeled for the 1–6 month (yellow), 7–12 month (orange), and 13–24 month periods.

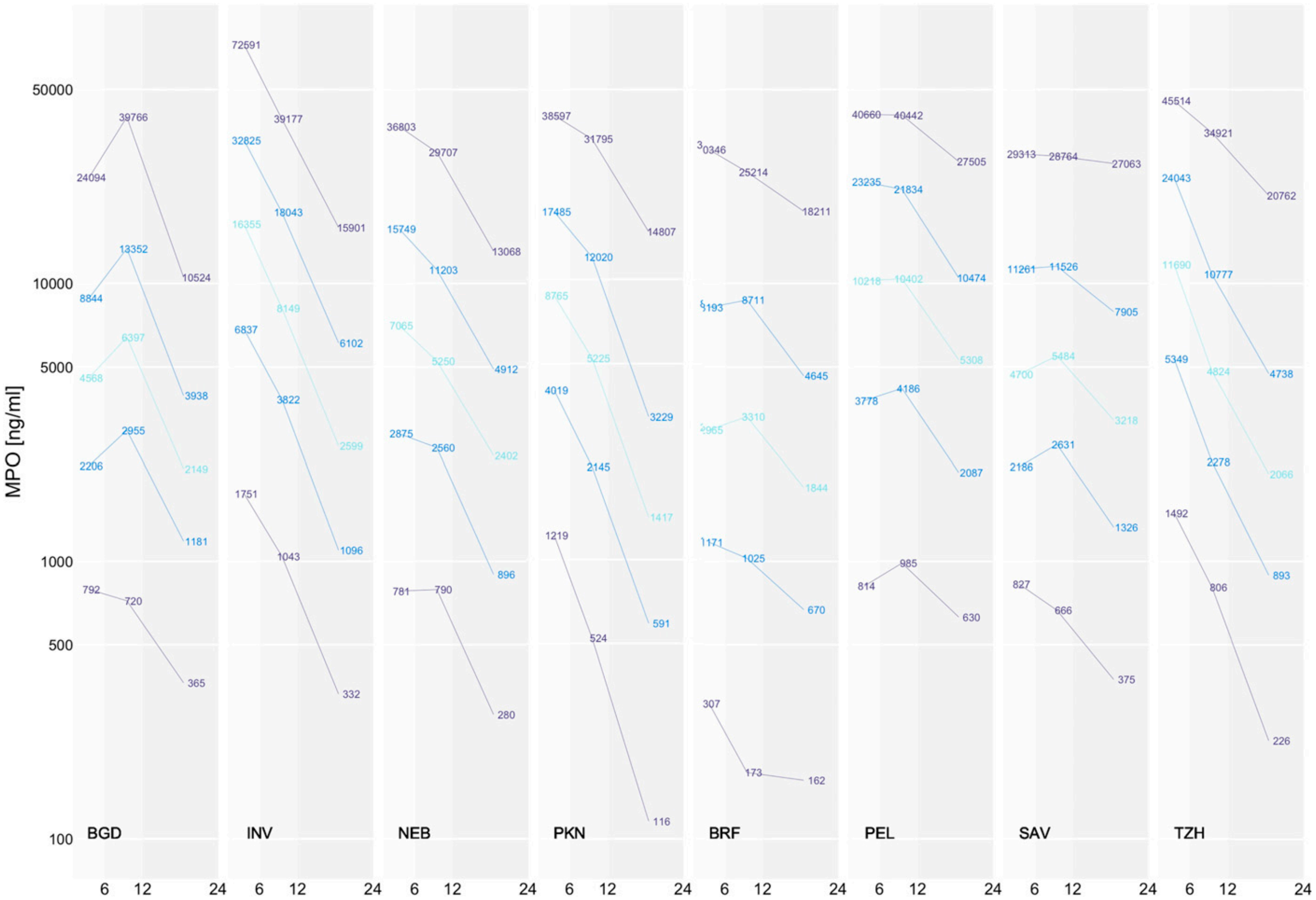

Supplemental Figure 2. The observed median (light blue), 25th–75th% range (midblue), and 5th–95th% range (dark blue) concentration of myeloperoxidase (MPO) at each site pooling the 1–6 month samples, the 7–12 month samples, and the 13–24 month samples.

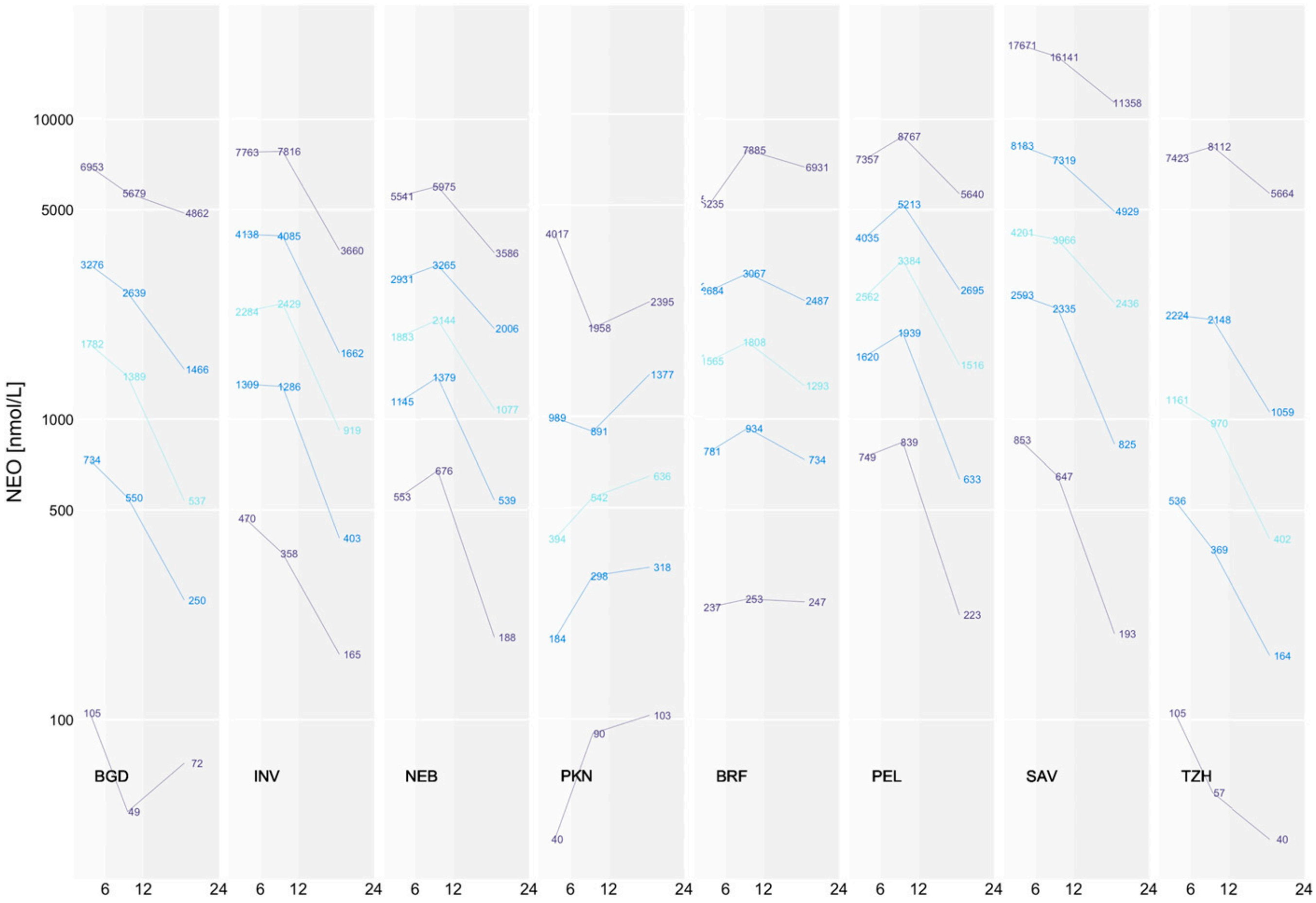

Supplemental Figure 3. The observed median (light blue), 25th–75th% range (midblue), and 5th–95th% range (dark blue) concentration of neopterin (NEO) at each site pooling the 1–6 month samples, the 7–12 month samples, and the 13–24 month samples.

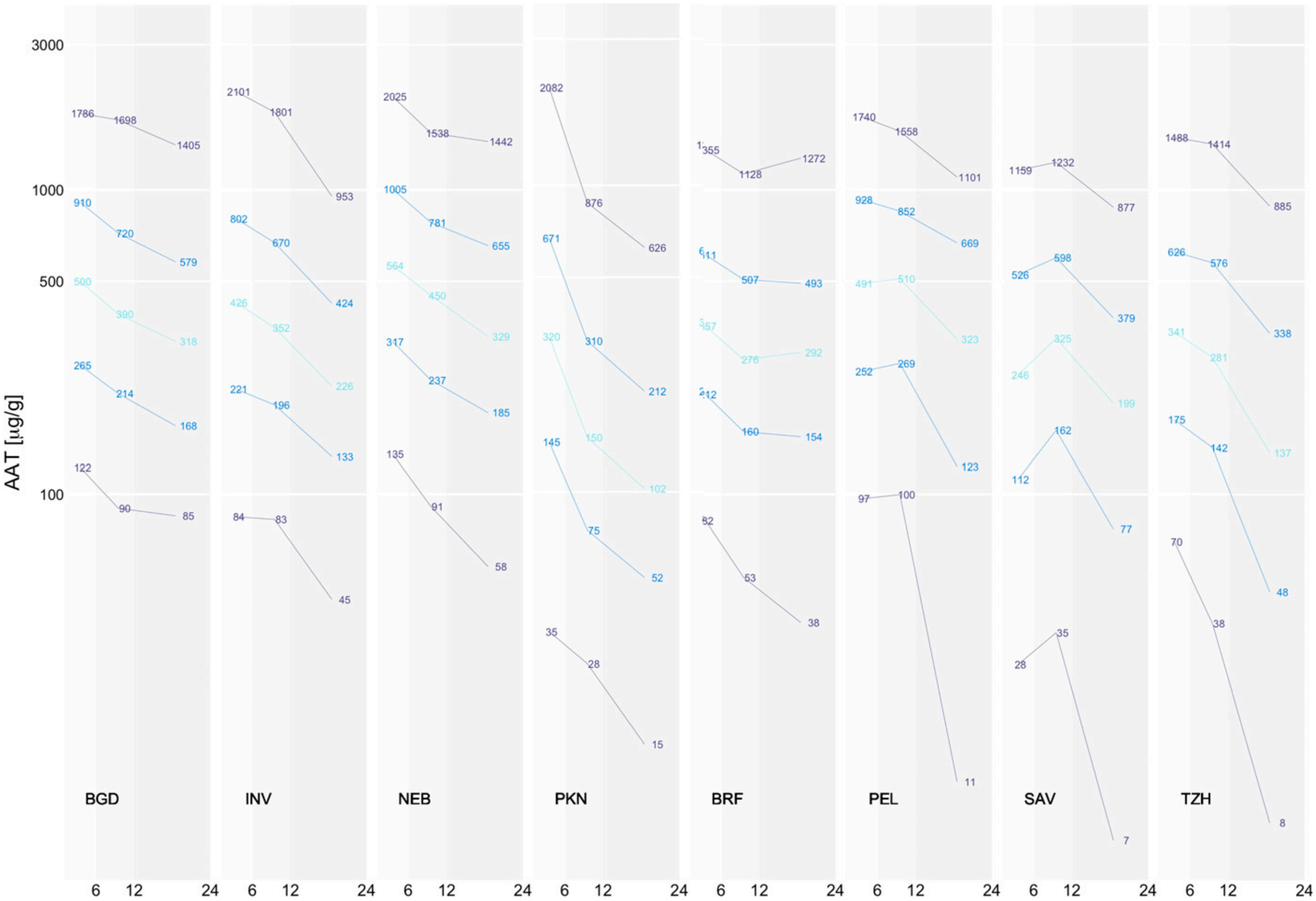

Supplemental Figure 4. The observed median (light blue), 25th–75th% range (midblue), and 5th–95th% range (dark blue) concentration of α-1-antitrypsin (AAT) at each site pooling the 1–6 month samples, the 7–12 month samples, and the 13–24 month samples.

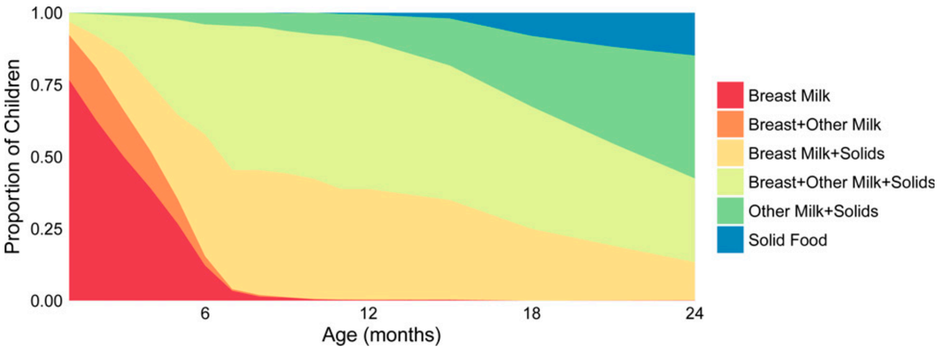

Supplemental Figure 5. The pattern of feeding in the 7 days preceding monthly stool samples. For the first 6 months, children tended to receive breast milk with or without additional foods. After 6 months, solid complementary foods were increasingly common and children began to be weaned off breast milk such that by 24 months, approximately 50% of children did not receive breast milk in the 7 days preceding the stool samples, although they did receive alternative animal milk.

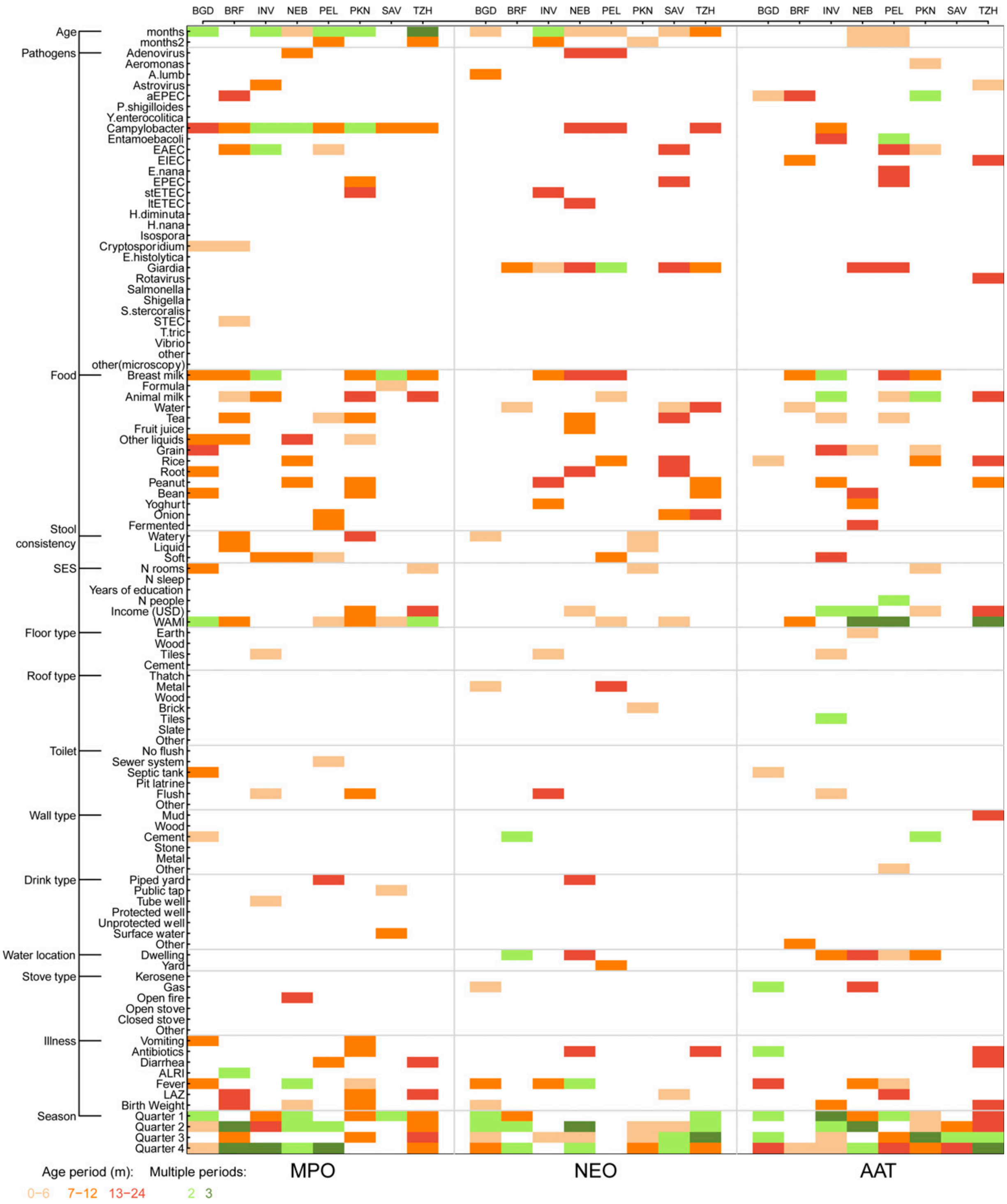

Supplemental Figure 6. Variables selected through the stochastic search variable selection (SSVS) with probability ≥ 0.65 in each of the biomarker models in each site. The brown color gradient indicates variables selected for a single age period (light brown, 0–6 months; orange, 7–12 months; or dark brown, 13–24 months) with probability of inclusion ≥ 0.65. Where variables were selected for two or all three age periods, they are shown in shades of green (light green, two ages; dark green, all three age groups). Variables with larger magnitude coefficients were more likely to be selected, whereas a low probability of selection reflects coefficients that can be approximated with a zero mean effect. Age and seasonal variables (the year quarter) were consistently selected for all sites and each of the biomarkers. The water and sanitation, assets, maternal education, and income (WAMI) index was more informative than individual factors that contribute to an understanding of socioeconomic status (SES) and breast milk was frequently selected. *Campylobacter* was selected in the myeloperoxidase (MPO) models (and associated with increased MPO concentrations), and *Giardia* was consistently selected to describe the neopterin (NEO) concentration (and associated with decreased concentrations). Symptoms of illness were inconsistently selected to describe the biomarkers between the eight sites.

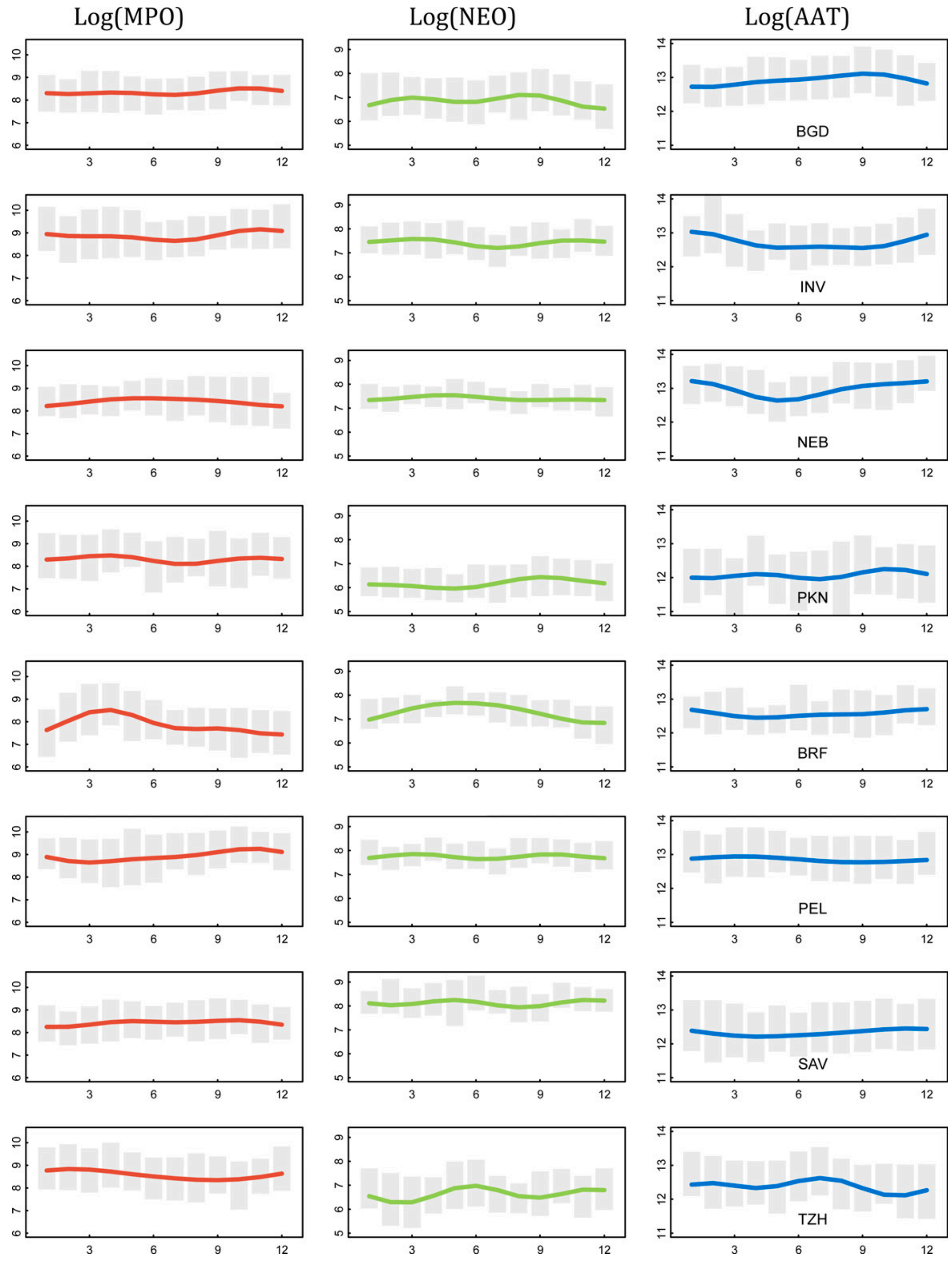

Supplemental Figure 7. Mean seasonality of the log concentrations of myeloperoxidase (MPO; red), neopterin (NEO; green), and α-1-antitrypsin (AAT; blue) at each site (rows). Grey bars indicate the interquartile range of each biomarker and lines indicate the mean fitted seasonal pattern.

Supplemental Table 1

Mean and SD for $\log_e$ concentrations of MPO, NEO, and AAT biomarkers pooled across sites by stool type (monthly, diarrhea, or inconclusive) and qualitative stool consistency

	Consistency	Inconclusive		Monthly		Diarrhea	
		n	Mean (SD)	*n*	Mean (SD)	*n*	Mean (SD)
MPO, ng/mL	Formed	799	8.18 (1.41)	5,003	8.13 (1.33)	305	8.25 (1.33)
	Soft	3,011	8.27 (1.53)	17,755	8.57 (1.35)	3,269	8.55 (1.42)
	Liquid	641	8.7 (1.31)	1,507	8.78 (1.28)	4,129	8.61 (1.48)
	Watery	272	8.82 (1.19)	252	8.83 (1.24)	1,650	8.83 (1.25)
NEO, nmol/L	Formed	799	7.41 (1.19)	5,003	7.37 (1.33)	305	7.38 (1.16)
	Soft	3,011	6.99 (1.32)	17,755	7.26 (1.27)	3,269	7.15 (1.23)
	Liquid	641	6.77 (1.55)	1,507	6.59 (1.33)	4,129	7.01 (1.23)
	Watery	272	7.04 (1.00)	252	7.46 (1.11)	1,650	7.31 (1.02)
AAT, μg/g	Formed	799	12.1 (1.38)	5,003	12.44 (1.17)	305	12.46 (1.05)
	Soft	3,011	12.5 (1.09)	17,755	12.64 (1.12)	3,269	12.25 (1.12)
	Liquid	641	12.19 (1.11)	1,507	12.46 (1.15)	4,129	12.1 (1.07)
	Watery	272	11.92 (1.35)	252	12.47 (1.17)	1,650	12.07 (1.09)

AAT = α-1-antitrypsin; MPO = myeloperoxidase; NEO = neopterin; SD = standard deviation. Significant differences between monthly and diarrheal stools were tested with two-tailed *t* tests. Stools classed as inconclusive were those collected according to the monthly schedule that during or close to episodes of diarrhea.
